# A Case of Severe Post‐Biopsy Bleeding and Perforation in Gastric Amyloidosis

**DOI:** 10.1002/jgh3.70065

**Published:** 2024-12-03

**Authors:** Sho Matsuyama, Akihisa Fukuda, Go Yamakawa, Taro Ueo, Hiroshi Seno

**Affiliations:** ^1^ Department of Gastroenterology and Hepatology Graduate School of Medicine, Kyoto University Kyoto Japan; ^2^ Department of Gastroenterology Tenri Hospital Nara Japan

**Keywords:** clip hemostasis, dialysis‐related amyloidosis, gastric amyloidosis, gastrointestinal amyloidosis

## Abstract

We herein describe a case of severe post‐biopsy bleeding and perforation in gastric amyloidosis. A 70‐year‐old man who had been on dialysis underwent esophagogastroduodenoscopy and biopsy was performed. Post‐biopsy bleeding occurred, and three times of coagulation hemostasis and once clip hemostasis were performed. After the hemostasis, he eventually had a gastric perforation and omental patch surgery was performed, however, he passed away after the surgery. From the pathological finding of biopsy specimen, he was diagnosed with gastric amyloidosis caused by dialysis‐related amyloidosis. In patients of amyloidosis, the risk of bleeding and perforation is elevated due to vascular and tissue fragility. Therefore, when performing hemostasis in patients with gastrointestinal amyloidosis, clip hemostasis which minimizes tissue damage is considered preferable to coagulation hemostasis.

**Abbreviations**
β2MG: beta‐2 microglobulin
DRA: dialysis‐related amyloidosis
EGD: esophagogastroduodenoscopy
HE: Hematoxylin and Eosin

A 70‐year‐old man underwent esophagogastroduodenoscopy (EGD) and biopsy was performed for a pale area near the Billroth‐I anastomosis after distal gastrectomy due to a duodenal ulcer. (Figure 1a,b). He had been on dialysis. Three days after EGD, he had hematemesis, and EGD showed an exposed blood vessel at the biopsy site (Figure 1c). Coagulation hemostasis was performed to the exposed blood vessel (Figure 1d), however, recurrent bleeding occurred on the following day (Figure 1e). Although coagulation hemostasis was performed for three times (Figure 1d–f), bleeding from the margin of ulcer occurred again (Figure 1g) and clip hemostasis was performed (Figure 1h). After clip hemostasis, no further bleeding was observed. Ten days after these hemostasis, he had a gastric perforation and omental patch surgery was urgently performed. Unfortunately, his overall condition was deteriorated, and he passed away 7 days after the surgery. The biopsy specimen revealed homogeneous and structure less eosinophilic substances by Hematoxylin and Eosin (HE) staining (Figure 2). The substances were positive for Congo‐red staining, Dylon staining, and immunostaining of beta‐2 microglobulin (β2MG) (Figure 2). Therefore, he was diagnosed with gastric amyloidosis caused by dialysis‐related amyloidosis (DRA).

**FIGURE 1:**
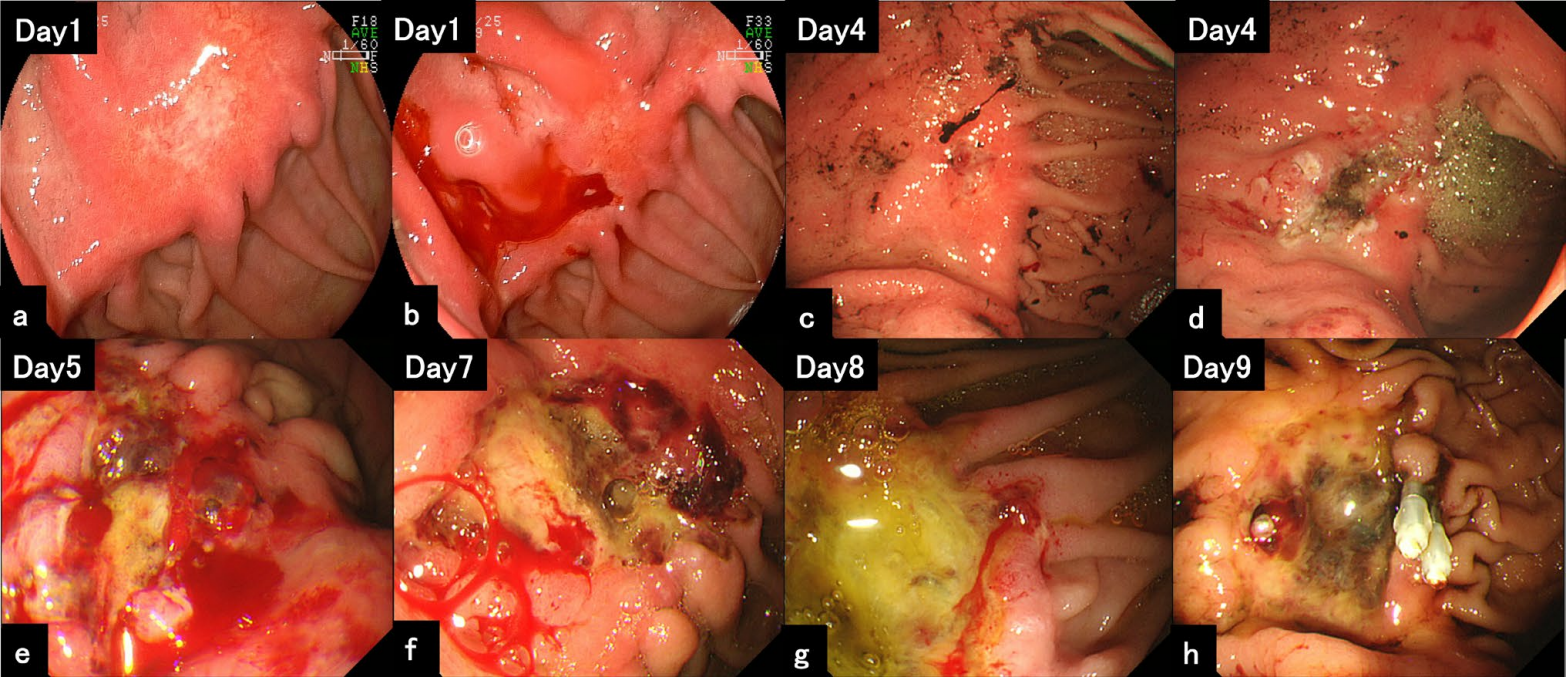
EGD images. (a–h) Day 1 to Day 9.

**FIGURE 2:**
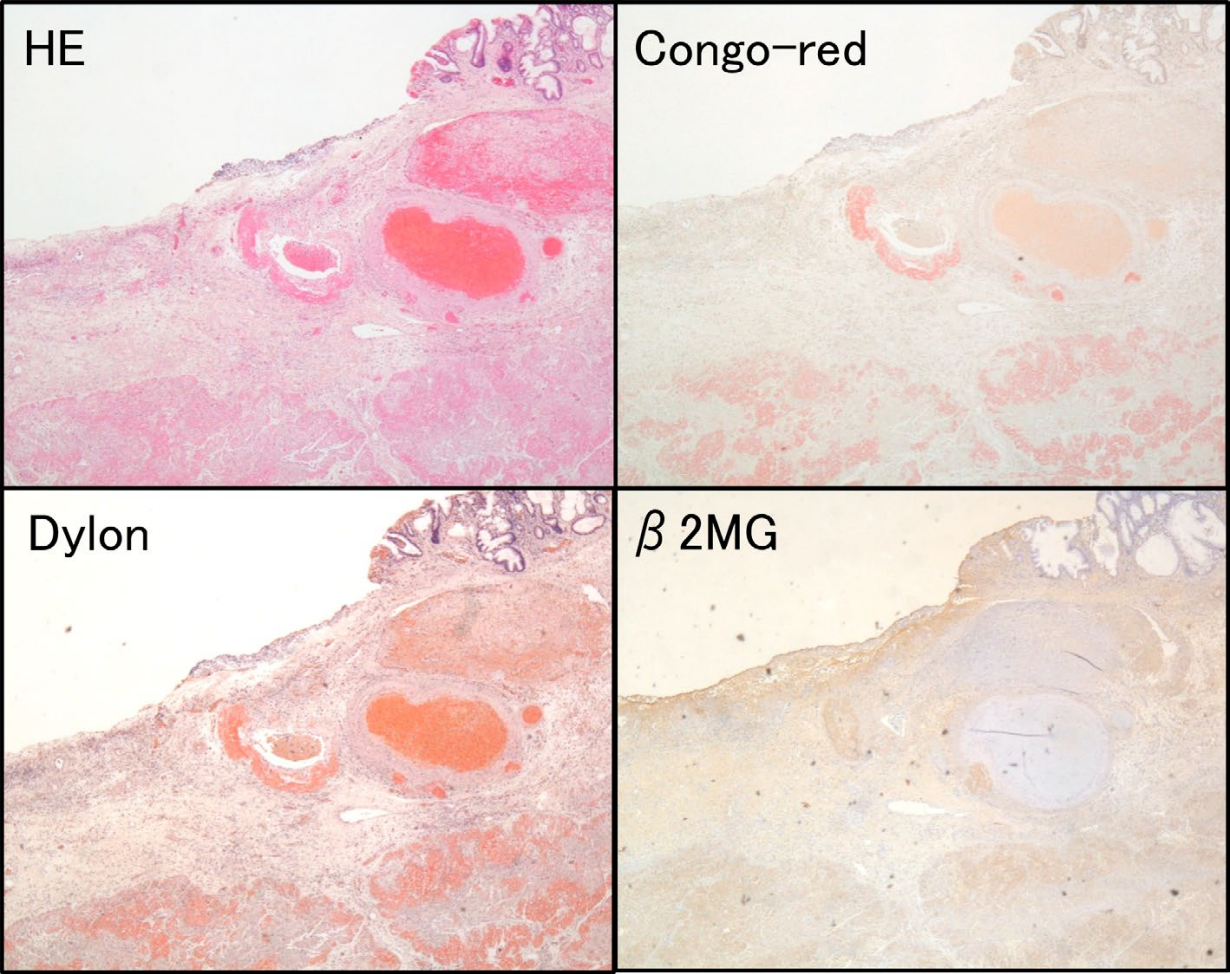
HE staining, Congo‐red staining, Dylon staining, and immunostaining of β2MG for biopsy specimen.

Amyloidosis is characterized by the extracellular deposition of amyloid proteins, affecting various organs, including the gastrointestinal tract. Amyloidosis includes several types, such as serum amyloid A amyloidosis, immunoglobulin light chain amyloidosis, dialysis‐related amyloidosis, hereditary amyloidosis, and wild‐type transthyretin amyloidosis. The pathophysiology of gastrointestinal bleeding from amyloidosis involves local ischemia, infarction, and mucosal injury that cause erosions, hematomas, or ulcerations [1]. There are no treatment guidelines for endoscopic therapy for bleeding from gastrointestinal amyloidosis, and the decision on which endoscopic therapy is used is based on the type of lesion and the endoscopist's preference [1]. Endoscopic hemostasis is often ineffective and surgical intervention may be required [2].

In the management of gastrointestinal bleeding, two commonly used methods of hemostasis are coagulation and clip hemostasis. The advantages of coagulation hemostasis are that coagulation can quickly stop bleeding by directly cauterizing and that it is effective for treating large bleeding areas or multiple small bleeding points. The advantages of clip hemostasis are that clips mechanically close the blood vessel to avoid thermal injury to the tissue and that it is suitable for fragile tissues with a lower risk of perforation.

In this case, the patient experienced bleeding because of a biopsy that was performed prior to the diagnosis of amyloidosis. Repeated coagulation was required for hemostasis, which might have contributed to the gastric perforation. DRA is reported to occur in approximately 20%–50% of long‐term dialysis patients, and it is important to keep in mind the possibility of DRA when performing biopsy or hemostasis for gastrointestinal bleeding in long‐term dialysis patients. The same caution is also necessary for patients with underlying conditions associated with amyloidosis other than DRA. In our case, there was a tissue damage by coagulation hemostasis, which not only exacerbated the bleeding but also might have contributed to gastric perforation. In patients of amyloidosis or in patients who have underlying conditions with amyloidosis, the risk of bleeding and perforation is elevated due to vascular and tissue fragility [3]. Clip hemostasis is considered preferable to coagulation hemostasis in cases in which the tissue is fragile, including cases of definitive amyloidosis as well as cases in which amyloidosis is suspected. Therefore, when performing hemostasis in patients with gastrointestinal amyloidosis, clip hemostasis which minimizes tissue damage is considered preferable to coagulation hemostasis. Furthermore, in cases of amyloidosis in which it is difficult to control bleeding with a risk of perforation, early consideration of surgical intervention is recommended before overall condition deteriorates severely.

## Ethics Statement

This case report was conducted in accordance with the ethical principles of the Declaration of Helsinki and approved by the Institutional Review Board of Tenri Hospital.

## Consent

To protect patient privacy, all data was anonymized, and personal information was handled in strict compliance with relevant regulations and guidelines. The data of this case report will be used solely for academic and research purposes.

## Conflicts of Interest

The authors declare no conflicts of interest.
